# Dual-energy CT with virtual monoenergetic images and iodine maps improves tumor conspicuity in patients with pancreatic ductal adenocarcinoma

**DOI:** 10.1186/s13244-022-01297-2

**Published:** 2022-09-24

**Authors:** Hongwei Liang, Yang Zhou, Qiao Zheng, Gaowu Yan, Hongfan Liao, Silin Du, Xiaohui Zhang, Fajin Lv, Zhiwei Zhang, Yong-mei Li

**Affiliations:** 1 Department of Radiology, The First Affiliated Hospital of Chongqing Medical University, No. 1 Youyi Road, Yuzhong District, Chongqing, 400016 China; 2 Department of Radiology, Suining Central Hospital, Suining, 629000 China

**Keywords:** Pancreatic cancer, Dual-energy computed tomography, Tumor conspicuity, Virtual monoenergetic images, Iodine maps

## Abstract

**Objectives:**

To evaluate the value of monoenergetic images (MEI [+]) and iodine maps in dual-source dual-energy computed tomography (DECT) for assessing pancreatic ductal adenocarcinoma (PDAC), including the visually isoattenuating PDAC.

**Materials and methods:**

This retrospective study included 75 PDAC patients, who underwent contrast-enhanced DECT examinations. Conventional polyenergetic image (PEI) and 40–80 keV MEI (+) (10-keV increments) were reconstructed. The tumor contrast, contrast-to-noise ratio (CNR) of the tumor and peripancreatic vessels, the signal-to-noise ratio (SNR) of the pancreas and tumor, and the tumor diameters were quantified. On iodine maps, the normalized iodine concentration (NIC) in the tumor and parenchyma was compared. For subjective analysis, two radiologists independently evaluated images on a 5-point scale.

**Results:**

All the quantitative parameters were maximized at 40-keV MEI (+) and decreased gradually with increasing energy. The tumor contrast, SNR of pancreas and CNRs in 40–60 keV MEI (+) were significantly higher than those in PEI (*p* < 0.05). For visually isoattenuating PDAC, 40–50 keV MEI (+) provided significantly higher tumor CNR compared to PEI (*p* < 0.05). The reproducibility in tumor measurements was highest in 40-keV MEI (+) between the two radiologists. The tumor and parenchyma NIC were 1.28 ± 0.65 and 3.38 ± 0.72 mg/mL, respectively (*p* < 0.001). 40–50 keV MEI (+) provided the highest subjective scores, compared to PEI (*p* < 0.001).

**Conclusions:**

Low-keV MEI (+) of DECT substantially improves the subjective and objective image quality and consistency of tumor measurements in patients with PDAC. Combining the low-keV MEI (+) and iodine maps may yield diagnostically adequate tumor conspicuity in visually isoattenuating PDAC.

## Key points

- Low-keV MEI (+) increases the image quality in patients with PDAC.
- Tumor conspicuity and reproducibility in tumor sizes were maximized at 40-keV MEI (+).
- Low-keV MEI (+) and iodine maps may achieve earlier diagnosis of isoattenuating PDAC.

## Introduction

Multidetector computed tomography (MDCT) is the most broadly used and preferred modality for the detection of pancreatic ductal adenocarcinoma (PDAC) [1]. However, early diagnosis remains a challenge, especially for the visually isoattenuating PDAC, which the tumor attenuation on contrast-enhanced CT is indistinguishable from the parenchymal attenuation [2–4]. Moreover, PDAC usually shows infiltrating growth and blurry margins, making it hard to accurately measure tumor size [5]. Accurate measurement of tumor size is crucially important for tumor staging and evaluation of treatment response such as radiotherapy [6–8].

Enhancing the contrast between the tumor tissue and pancreatic parenchyma could be expected to increase tumor conspicuity and potentially improve the reproducibility of tumor measurements [6]. Dual-energy CT (DECT) allows for tumor characterization and quantitation based on the unique absorption characteristics of each material at different energy levels [9]. Monoenergetic images (MEIs) acquired from different types of DECT (dual-source, rapid kV-switching, and dual-layer DECT) have been proven to improve tissue contrast [10, 11]. MEI approximates the image derived from monoenergetic X-rays, in which iodine attenuation increases with the energy approaching the K-edge of iodine (33.2 keV) [12, 13]. However, it is reported that due to the increased image noise at low keV levels, especially in abdominal examination, the clinical utility of MEI at < 70 keV is limited [5, 14].

Recently, a third-generation dual-source DECT has been introduced to overcome this limitation, which allows for the reconstruction of monoenergetic image plus (MEI [+]) by using a noise-optimized monoenergetic algorithm [13, 15]. Based on a spatial frequency-split recombination technique, both the higher-image contrast at lower energies and superior noise properties at higher energies are combined to optimize the image noise level [10, 16]. Several prior studies have shown that low-keV MEI (+) of DECT increased the pancreas-tumor contrast [10, 12, 16], but its utility for tumor measurements reproducibility and visually isoattenuating PDAC has been scantly investigated. Aside from reconstruction of MEI (+), iodine maps can also be obtained from DECT. Iodine maps are material decomposition images, which can quantitatively calculate the iodine concentration of the lesions [17, 18]. This indicates that iodine maps may be helpful to detect visually isoattenuating PDAC [3].

The purpose of this study was to assess the image quality, reproducibility of tumor measurements, and conspicuity on MEI (+) at different keVs, compared to polyenergetic images (PEI), in patients with PDAC. Additionally, we investigated whether MEI (+) and iodine maps could provide sufficient diagnostic confidence for the visually isoattenuating PDAC.

## Materials and methods

### Patient population

**Fig. 1:**
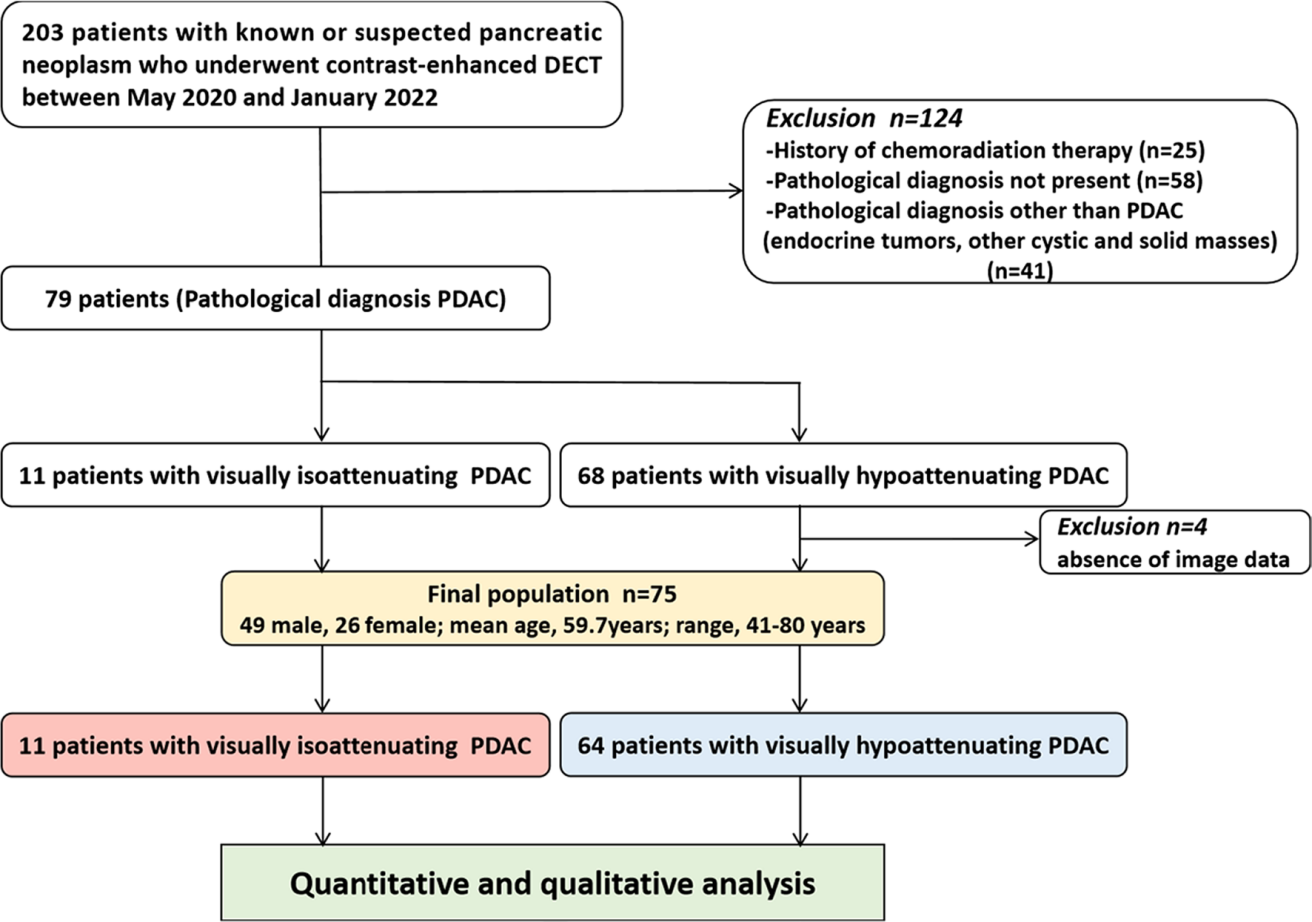
Flow chart for the inclusion and exclusion of patients and imaging characterization of study subjects

Our institutional review board approved this retrospective study and waived the need for written informed consent. Between May 2020 and January 2022, 203 consecutive patients with known or suspected pancreatic neoplasm underwent multiphasic CT scan on a 192-section third-generation dual-source DECT scanner (SOMATOM Force, Siemens Healthcare) for tumor assessment. Exclusion criteria included: no pathological diagnosis (*n* = 58) or diagnosed except PDAC like Islet cell tumor, other solid and cystic masses (*n* = 41), history of chemoradiation therapy (*n* = 25), and absence of image data (*n* = 4). Finally, 75 patients with definite histologically diagnosis of PDAC by pancreatectomy (*n* = 33), percutaneous biopsy (*n* = 19), or endoscopic biopsy (*n* = 23) were included in the study. Detailed inclusion and exclusion criteria are shown in Fig. 1.

### Image acquisition

All CT scans were obtained with the patient in the head first supine position with a dual-source DECT scanner using the following parameters: tube A, 100 kV/260 ref.mAs; tube B, Sn 150 kV/130 ref.mAs with tin filter; collimation, 128 × 0.6 mm; rotation time, 0.5 s; and pitch, 0.6. Automatic tube current modulation (CAREDose4D) was activated by default. After acquiring non-contrast CT images, a non-ionic contrast media (Iopamiro 370, Bracco Healthcare; or Ultravist 370, Bayer Healthcare) was intravenously injected with an iodine concentration of 450 mg iodine per kilogram and at a rate of 3.5–5.0 mL/s. Arterial, pancreatic parenchymal and portal venous phase scans started at 10, 23, and 48 s, respectively, after the predetermined threshold reached 100 HU in the abdominal aorta.

The scanner generated automatically linear-blended images with a blending factor of 0.6 (M 0.6, combining 60% of the 100 kV with 40% of the Sn150 KV spectrum) to represent the standard 120 kV impression. Noise-optimized MEI (+) was reconstructed at 40-, 50-, 60-, 70-, and 80-keV levels on a syngovia workstation (syngo.via, version VB20A; Siemens Healthineers). Iodine maps of the pancreatic parenchymal phase were reconstructed to calculate iodine concentration (in mg/mL). All images were reformatted as axial and coronal orientation with a thickness of 1 mm and an increment of 1 mm.

### Quantitative analysis

All CT images were quantitatively evaluated by one resident radiologist with 6 years of abdominal imaging experience on a syngovia workstation. For corresponding enhancement phase images, circular regions of interest (ROIs) were defined in the following areas: tumor tissue, normal pancreatic parenchyma, peripancreatic arteries (common hepatic artery [CHA], celiac trunk [CT], and superior mesenteric artery [SMA]), peripancreatic veins (portal vein [PV] and superior mesenteric vein [SMV]), and subcutaneous fat. CT attenuations of different regions were measured in Hounsfield units (HU); image noise was recorded as the standard deviation (SD) of subcutaneous fat. For parenchyma and tumor ROIs, care was taken to avoid inclusion of the calcification, vessels, visible pancreatic duct, and necrotic collections. For vessel analysis, the ROIs were as large as possible but did not include the vascular wall and calcification. All measurements were taken twice by the resident radiologist to ensure data consistency, and the mean values were used for analysis. The accuracy of ROIs placement was confirmed by a senior radiologist with 15 years of experience. The quantitative image quality of each object is calculated with Eq. [10]:

$$\begin{aligned} & {\text{Tumor}}\,{\text{contrast}} = {\text{HU}}_{{{\text{pancreas}}}} - {\text{HU}}_{{{\text{tumor}}}} \\ & {\text{CNR}}_{{{\text{tumor}}}} = ({\text{HU}}_{{{\text{pancreas}}}} - {\text{HU}}_{{{\text{tumor}}}} ){\text{/SD}}_{{{\text{fat}}}} \\ & {\text{SNR}}_{{{\text{tumor}}}} = ({\text{HU}}_{{{\text{tumor}}}} ){\text{/SD}}_{{{\text{fat}}}} \\ & {\text{SNR}}_{{{\text{pancreas}}}} = ({\text{HU}}_{{{\text{pancreas}}}} ){\text{/SD}}_{{{\text{fat}}}} \\ & {\text{CNR}}_{{{\text{vessel}}}} = ({\text{HU}}_{{{\text{vessel}}}} - {\text{HU}}_{{{\text{pancreas}}}} ){\text{/SD}}_{{{\text{fat}}}} \\ \end{aligned}$$

For the tumor measurements, two radiologists with 6 and 15 years of abdominal imaging experience who were blinded to the initial energy settings independently measured the maximal long and short diameters of the tumor in the axial pancreatic parenchyma phase. Material-specific iodine maps were reconstructed to quantitatively assess the iodine uptake of the tumor. To minimize variations in circulation and scanning time among individuals, normalized iodine concentration (NIC) was calculated by the ratio of iodine concentration in the tumor to that of the abdominal aorta (NIC_tumor_ = IC_tumor_/IC_aorta_ and NIC_parenchyma_ = IC_parenchyma_/IC_aorta_). Tumors visually presented isoattenuating and hypoattenuating on PEI image were classified into two subgroups. Based on the Seong Ho Park’ study [2], visually isoattenuating PDAC was defined as the following criterion: (a) Compared with normal pancreatic parenchyma, no pancreatic lesions with visually attenuation changes were observed on contrast-enhanced CT. (b) No CT findings of serious obstructive pancreatitis (pancreatitis caused by tumor obstructed the pancreatic duct) or focal pancreatitis were found. If no mass was observed, the tumor was the location of interrupted pancreatic or common bile duct or contour abnormality of the pancreas. For the visually isoattenuating PDAC, we also reviewed all available MR images and checked patients’ surgical and pathological reports, which described the precise location of the tumors, before the quantitative analysis.

### Qualitative analysis

The same two radiologists, who were unaware of reconstruction protocol, independently and randomly evaluated all MEI (+) and PEI images. Five-point scale was used in assessing tumor conspicuity (1, non-diagnostic; 2, indeterminate diagnosis; 3, fair diagnosis; 4, clear diagnosis; and 5, definite diagnosis), tumor edge sharpness (1, poor; 2, fair; 3, adequate; 4, sharp; and 5, sharpest), image quality (1, unacceptable; 2, fair; 3, adequate; 4, good [clear anatomical structure, mild image noise]; and 5, excellent [quite clear anatomical structure, minimal image noise]), and the visualization of the peripancreatic vessels (CT, CHA, SMA, PV and SMV) (1, poor; 2, fair; 3, average; 4, good [vessel strengthened well]; and 5, excellent [vessel strengthened obviously, the vessel edge is sharp]) [12, 19]. The two observers’ mean scores were statistically analyzed.

### Statistical analysis

SPSS statistical software (version 26.0; IBM Cor, 2013) was used to perform all data analyses. The normality of data distribution was evaluated by the Kolmogorov–Smirnov test. Continuous variables were displayed as means ± SD. The quantitative parameters in tumor contrast, CNRs and SNRs were compared using analysis of variance (ANOVA) test and the Dunnett correction. Kruskal–Wallis test with Steel–Dwass post hoc correction were used to compare the image noise, tumor measurements and qualitative parameters. The Mann–Whitney U test was used to compare the iodine concentration between tumor and normal pancreatic parenchyma. Intra-observer variability in tumor measurements was evaluated via the intraclass correlation coefficient (ICC). The interobserver agreement for subjective analyses was assessed using kappa coefficients, with a *k* value of ≤ 0.20 indicating poor agreement, 0.21–0.40 fair, 0.41–0.60 moderate, 0.61–0.80 good, and ≥ 0.81 excellent agreement. *P* values less than 0.05 were considered to indicate a statistically significant difference.

## Results

### Study population

Seventy-five patients (49 males, 26 females; mean age, 59.7 ± 8.1 years; range, 41–80 years) with histologically proven PDAC were included in the study. Of those, 25 patients (33.33%) were resectable PDAC, 8 (10.67%) were borderline resectable PDAC, 30 (40.0%) were local advanced PDAC, and 26 (34.67%) had distant metastasis. The tumors shown as visually hypoattenuating were found in 64 patients (85.3%) and isoattenuating in 11 patients (14.7%) on PEI images.

### Quantitative image analysis

Table 1 summarizes the results of quantitative analyses. For all patients, all quantitative parameters were maximal at 40-keV MEI (+) and decreased gradually with increasing energy. The tumor contrast, CNR of the tumor and vessels in 40–60 keV MEI (+) were significantly higher than those in PEI (*p* < 0.05). For pancreatic parenchyma, 40–60 keV MEI (+) provided significantly higher SNR compared with PEI (*p* < 0.01). In contrast to the SNR of pancreas, there were no significant differences in SNR of tumor between PEI and MEI (+) (*p* = 0.167). Image noise in 40–60 keV MEI (+) was significantly higher than that in PEI (*p* < 0.01).

**Table 1:** Quantitative analysis

	40 keV	50 keV	60 keV	70 keV	80 keV	PEI
Image noise (HU)	*23.8 ± 2.3	*19.0 ± 2.2	*16.1 ± 1.9	13.6 ± 1.7	12.8 ± 1.7	12.9 ± 1.6
Tumor contrast (HU)	*177.0 ± 62.2	*117.6 ± 41.8	*81.6 ± 29.5	56.5 ± 21.3	45. 7 ± 18.0	49.3 ± 19.6
*CNR*						
PDAC	*7.4 ± 2.6	*6.2 ± 2.2	*5.1 ± 1.9	4.2 ± 1.6	3.6 ± 1.4	3.8 ± 1.5
CA	*35.0 ± 8.9	*28.9 ± 7.5	*23.5 ± 5.8	19.4 ± 5.3	15.6 ± 4.0	17.3 ± 4.6
CHA	*31.1 ± 7.9	*26.2 ± 6.8	*21.1 ± 5.0	18.4 ± 4.5	14.6 ± 3.6	16.2 ± 4.4
SMA	*34.0 ± 9.0	*28.3 ± 7.4	*22.8 ± 5.6	19.3 ± 5.0	15.3 ± 3.9	16.9 ± 4.6
PV	*10.3 ± 4.5	*8.5 ± 4.0	*6.7 ± 3.1	5.9 ± 2.6	4.5 ± 2.2	5.3 ± 2.4
SMV	*9.4 ± 4.7	*7.6 ± 3.8	*6.1 ± 3.1	5.2 ± 2.6	3.9 ± 2.1	4.7 ± 2.6
*SNR*						
PDAC	5.5 ± 3.2	5.2 ± 2.8	4.8 ± 2.4	4.7 ± 2.1	4.5 ± 1.9	4.9 ± 2.0
Pancreas	*12.9 ± 2.7	*11.3 ± 2.5	*9.9 ± 2.2	8.9 ± 1.9	8.0 ± 1.8	8.7 ± 1.6

Data are expressed as mean ± SD

PDAC, pancreatic ductal adenocarcinoma; HU, Hounsfield units; tumor contrast, HU_pancreas _− HU_tumor_; CNR, contrast-to-noise ratio; SNR, signal-to-noise ratio; CA, celiac artery; CHA, common hepatic artery; SMA, superior mesenteric artery; PV, portal vein; SMV, superior mesenteric vein

*MEI (+) > PEI in the quantitative image quality (*p* < 0.05)

**Fig. 2:**
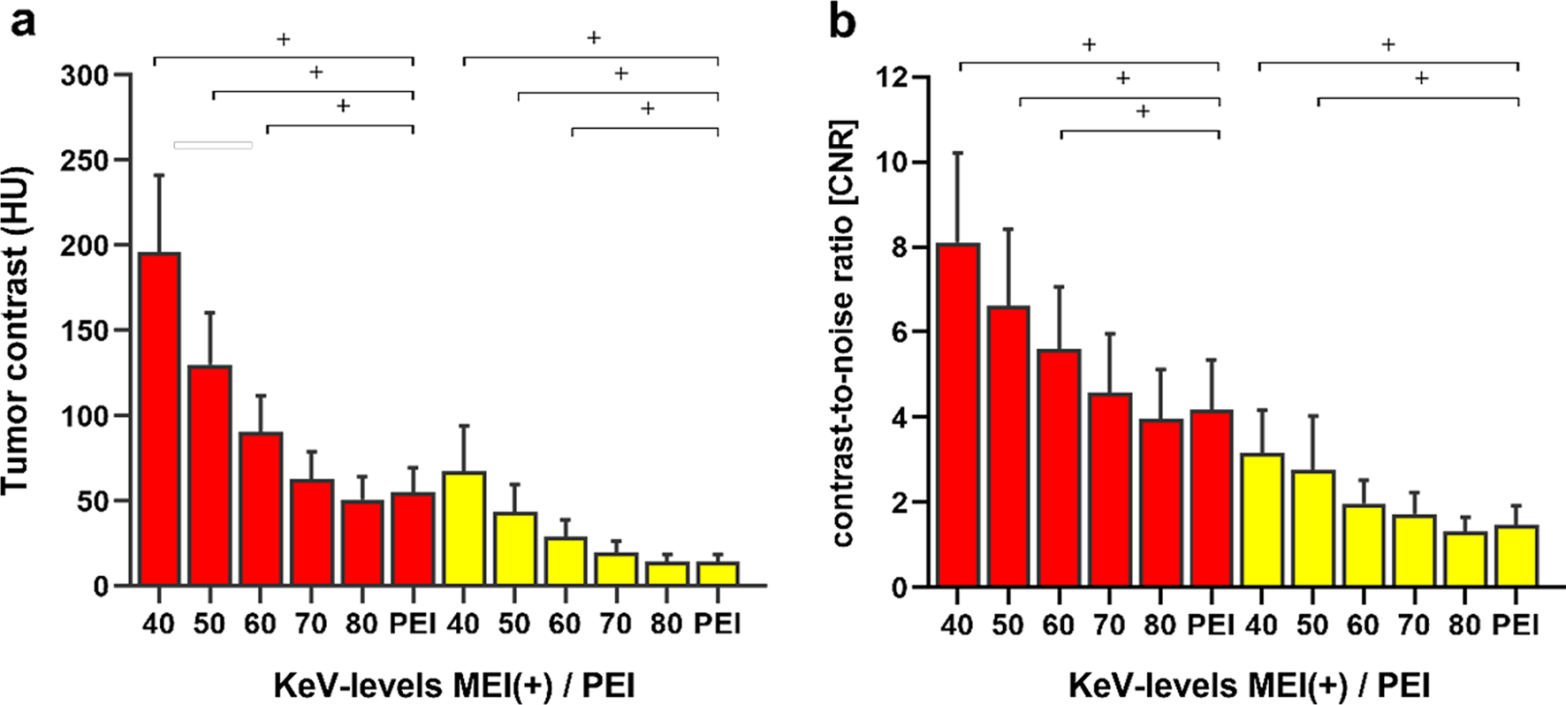
Bar graph showing the tumor contrast (**a**) and contrast-to-noise ratio (CNR) (**b**) between tumor and normal parenchyma in the visually hypoattenuating (red bars) and isoattenuating (yellow bars) pancreatic ductal adenocarcinoma (PDAC). ^+^MEI (+) > PEI in the objective image characteristics (*p* < 0.05)

For visually hypoattenuating and isoattenuating PDAC separated as subgroups, the tumor contrast (Fig. 2a) and CNR of tumor (Fig. 2b) presented similar results, with the maximum values in 40-keV MEI (+), followed by 50 keV. The tumor contrast and CNR of tumor in 40–60 keV MEI (+) of visually hypoattenuating PDAC were significantly higher than those in PEI (*p* < 0.001). The tumor contrast in 40–60 keV MEI (+) and CNR of tumor in 40–50 keV MEI (+) of visually isoattenuating PDAC were significantly higher than those in PEI (*p* < 0.05).

The measurements of the maximal long and short diameters of hypoattenuating and isoattenuating PDAC are summarized in Table 2. The range of visually hypoattenuating tumor measurements (mean of longest diameter ± SD, 32.6 ± 10.2 to 37.6 ± 11.3 mm) was higher than the isoattenuating tumors (26.2 ± 4.6 to 28.3 ± 4.7 mm). Inter-reader agreement of the measurements of maximal long and short diameters of tumors was excellent agreement (ICC, 0.969–0.993), and 40-keV MEI ( +) had the highest reproducibility between the two radiologists.

**Table 2:** Measurements of the maximal long and short diameters of PDAC

	Hypoattenuating tumor						Isoattenuating tumor					
	40 keV	50 keV	60 keV	70 keV	80 keV	PEI	40 keV	50 keV	60 keV	70 keV	80 keV	PEI
Long diameters (mm)												
R1	32.6 ± 10.2	32.9 ± 10.1	34.4 ± 10.2	35.6 ± 10.6	37.2 ± 11.0	35.8 ± 10.7	26.2 ± 4.6	26.7 ± 4.8	27.0 ± 4.6	27.5 ± 4.8	28.3 ± 4.7	27.7 ± 4.9
R2	32.9 ± 10.1	33.6 ± 10.0	34.9 ± 10.2	36.2 ± 10.9	37.6 ± 11.3	36.3 ± 10.9	26.5 ± 4.9	26.8 ± 5.0	27.2 ± 4.9	27.2 ± 4.9	28.1 ± 5.0	27.6 ± 4.7
ICC (95%CI)	0.991 (0.986–0.995)	0.986 (0.978–0.992)	0.986 (0.978–0.992)	0.982 (0.970–0.989)	0.975 (0.959–0.985)	0.984 (0.974–0.990)	0.989 (0.960–0.997)	0.987 (0.953–0.996)	0.978 (0.919–0.994)	0.979 (0.923–0.994)	0.969 (0.890–0.992)	0.976 (0.912–0.993)
Short diameters (mm)												
R1	24.0 ± 7.4	24.1 ± 7.3	25.4 ± 7.4	25.7 ± 7.7	26.2 ± 7.5	25.3 ± 7.3	21.9 ± 5.3	22.3 ± 5.4	23.3 ± 5.0	23.5 ± 5.0	24.1 ± 5.6	23.2 ± 5.4
R2	24.1 ± 7.5	24.2 ± 7.4	25.7 ± 7.5	25.6 ± 7.3	26.2 ± 7.5	25.4 ± 7.4	21.9 ± 5.5	22.4 ± 6.2	23.0 ± 5.7	23.0 ± 6.0	23.4 ± 5.6	22.9 ± 5.8
ICC (95%CI)	0.990 (0.983–0.994)	0.988 (0.981–0.993)	0.984 (0.974–0.990)	0.979 (0.965–0.981)	0.984 (0.973–0.990)	0.977 (0.962–0.986)	0.993 (0.974–0.998)	0.984 (0.943–0.996)	0.976 (0.913–0.993)	0.980 (0.928–0.995)	0.970 (0.892–0.992)	0.981 (0.931–0.995)

Data are expressed as mean ± SD

PDAC, pancreatic ductal adenocarcinoma; ICC, intraclass correlation coefficient; CI, confidence interval

### Iodine uptake

**Fig. 3:**
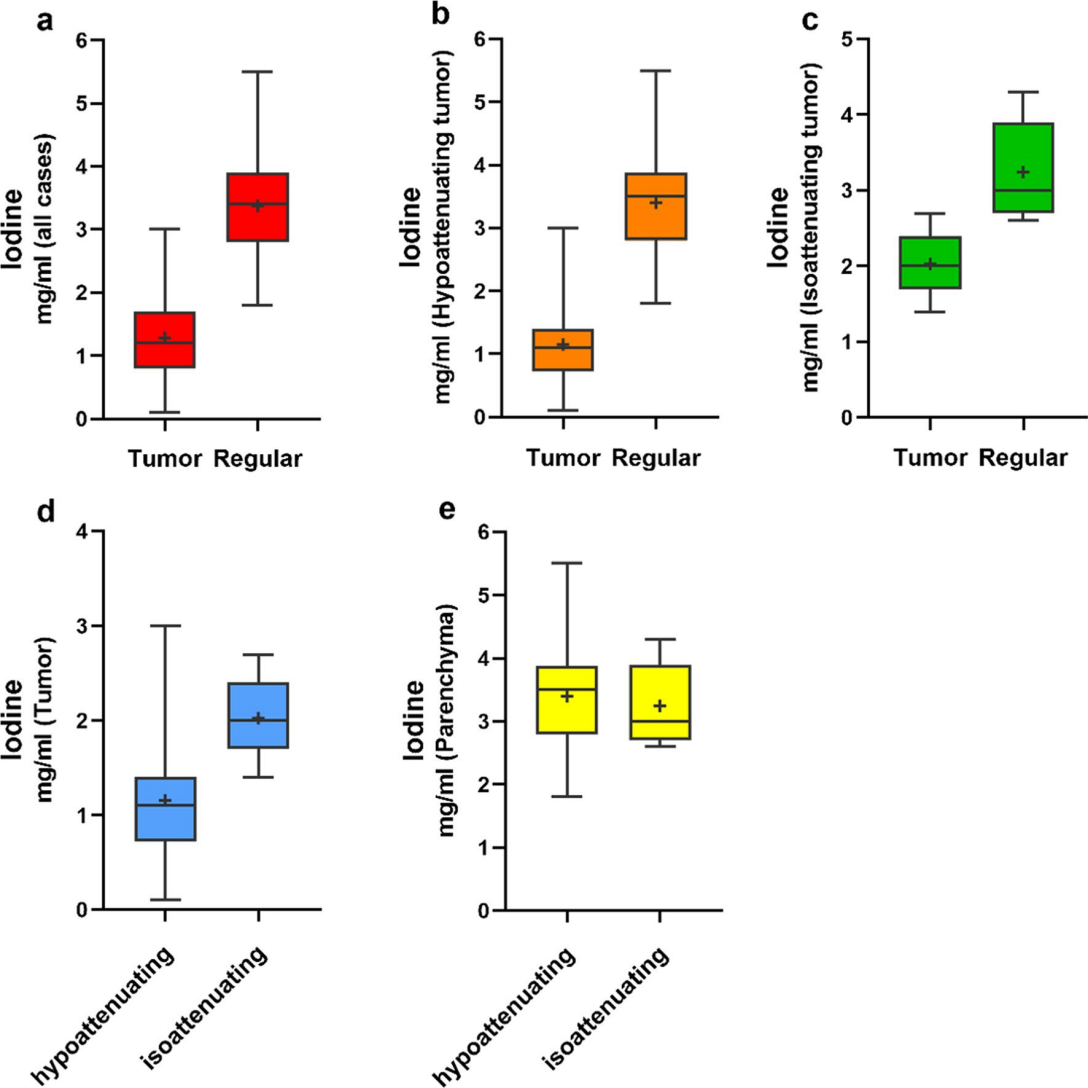
Normalized iodine concentration (NIC) was calculated and compared between tumor and regular pancreatic parenchyma (**a**–**c**). In panel **d**, **e**, the NIC between the visually hypoattenuating and isoattenuating pancreatic ductal adenocarcinoma (PDAC) is depicted. Box shows the 25-and 75-quartile, and horizontal line represents median value, and the cross indicates average value

For all cases, there was significantly lower NIC value of tumor tissue than the normal pancreatic parenchyma (1.28 ± 0.65 vs 3.38 ± 0.72 mg/mL; *p* < 0.001, Fig. 3a). Also, the NIC value of tumor was significantly lower than the parenchyma in the visually hypoattenuating PDAC (1.15 ± 0.59 vs. 3.40 ± 0.75 mg/mL; *p* < 0.001, Fig. 3b) and in the visually isoattenuating PDAC (2.03 ± 0.44 vs 3.25 ± 0.54 mg/mL, *p* < 0.001, Fig. 3c). Moreover, the NIC value of tumor in the visually hypoattenuating PDAC (1.15 ± 0.59 mg/mL) was significantly lower than the visually isoattenuating PDAC (2.03 ± 0.44 mg/mL) (*p* < 0.001, Fig. 3d). However, there were no significant differences in NIC value of parenchyma between visually hypoattenuating and isoattenuating PDAC (3.40 ± 0.75 mg/mL vs 3.25 ± 0.54 mg/mL; *p* = 0.481, Fig. 3e).

### Qualitative image analysis

**Fig. 4:**
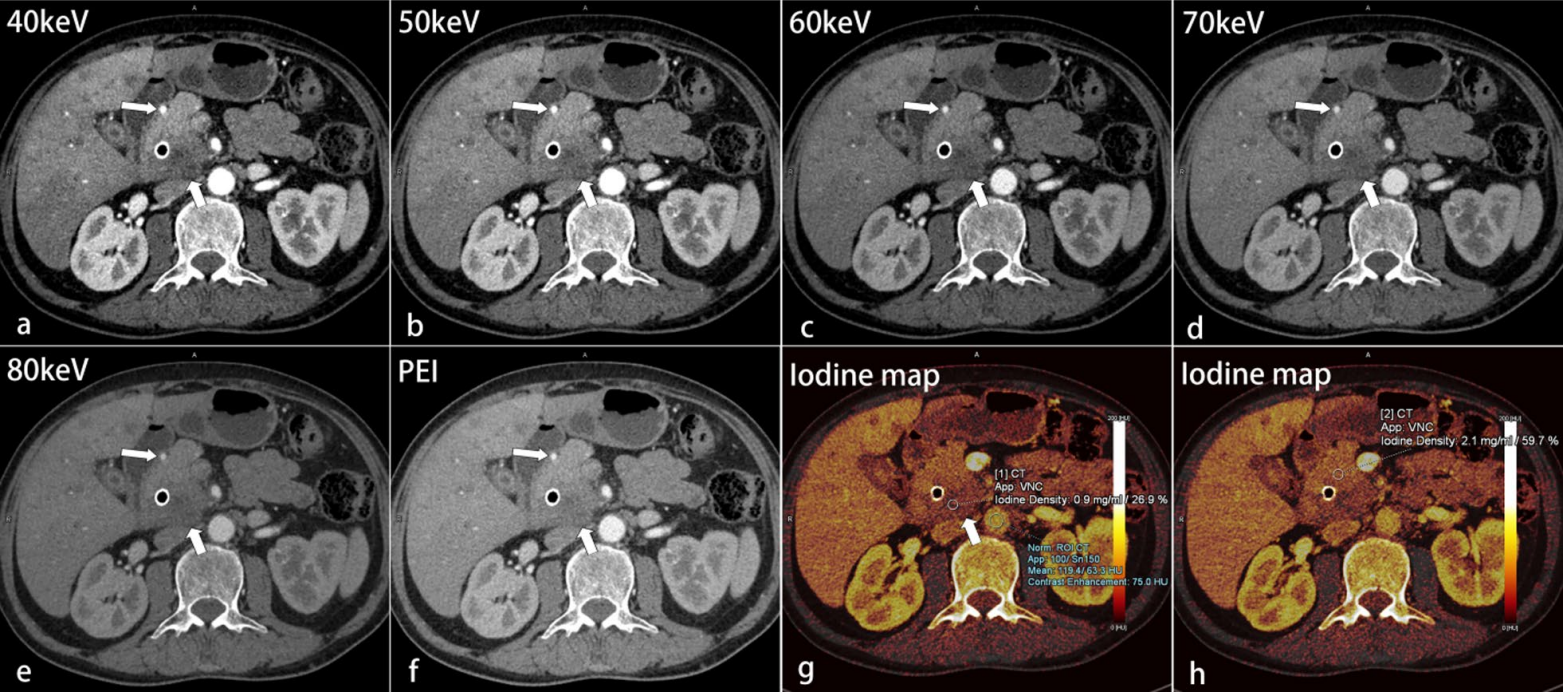
Transverse contrast-enhanced images of a 62-year-old man with hypoattenuating pancreatic ductal adenocarcinoma (PDAC) at the uncinate process of the pancreas (thick arrows) after bile duct stenting. The tumor conspicuity, margin delineation, and depiction of gastroduodenal artery (thin arrows) were best in the MEI (+) at 40 keV (**a**), followed by 50–80 keV (**b**–**e**), and were better than those in PEI (**f**). On iodine maps, the iodine concentrations quantified in the tumor (**g**) and pancreatic parenchyma (**h**) were normalized to the aorta (blue circle) as normalized iodine concentration (NIC). The NIC_tumor_ value of 0.9 mg/mL was significantly lower than the NIC_parenchyma_ value of 2.1 mg/mL

**Fig. 5:**
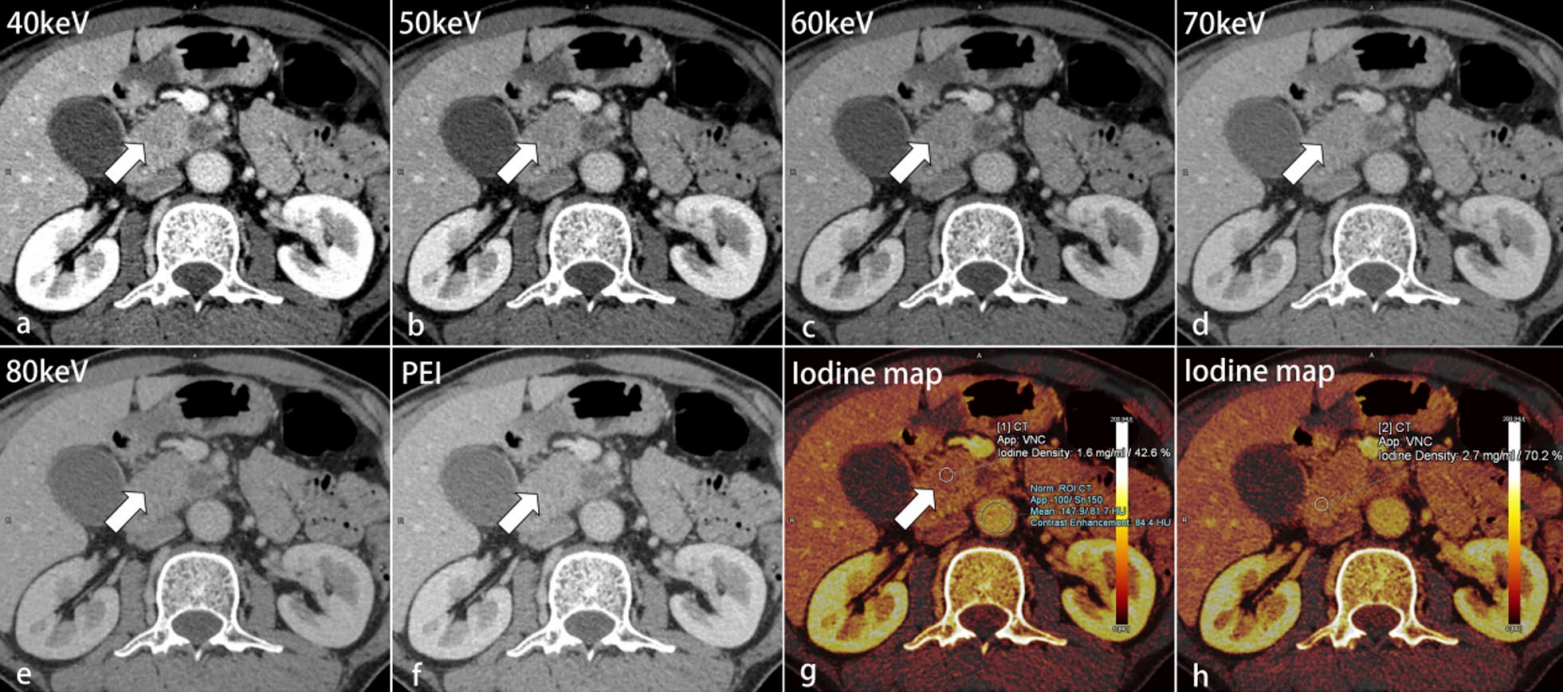
Transverse contrast-enhanced images of a 58-year-old man with visually isoattenuating pancreatic ductal adenocarcinoma (PDAC) at the pancreatic head (arrows). Tumor was difficult to detect in PEI (**f**), which showed a ill-defined mass. As the energies decreased (**a**–**e**), the contrast between the tumor and normal parenchyma appeared to improve. In this case, tumor presented a slightly low density area on iodine maps (**g**, **h**). The NIC_tumor_ value of 1.6 mg/mL was significantly lower than the NIC_parenchyma_ value of 2.7 mg/mL

**Fig. 6:**
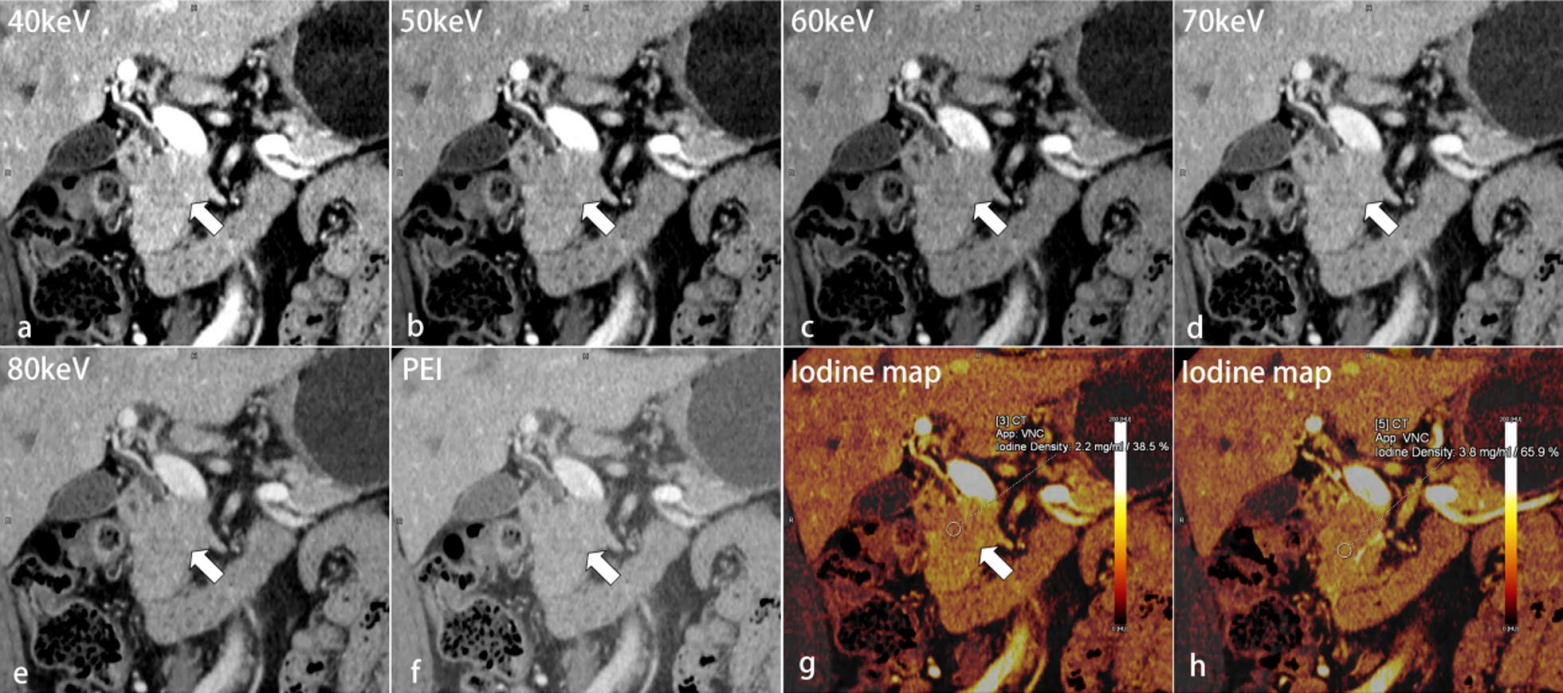
Coronal reformatted image of a 66-year-old man with visually isoattenuating pancreatic ductal adenocarcinoma (PDAC) at the pancreatic head (arrows). Conventional polyenergetic image (PEI) revealed an isoattenuating mass, which was difficult to distinguish from the normal parenchyma (**f**). The 40-keV MEI (+) (**a**) showed the improvement of tumor conspicuity and margin delineation compared with 50–80 keV MEI (+) and PEI (**b**–**f**). On iodine maps (**g**, **h**), the NIC_tumor_ value of 2.2 mg/mL confirmed the diagnosis of tumor, which was significantly lower than the NIC_parenchyma_ value of 3.8 mg/mL. Combining low-keV MEI (+) and iodine maps improved the tumor conspicuity of isoattenuating PDAC, possibly enabling the early diagnosis

Table 3 shows the results of qualitative analyses. For all cases, 40-keV MEI (+) provided the highest subjective scores for all criteria except image quality (Figs. 4, 5, 6). The subjective scores for tumor conspicuity, tumor margin sharpness and image quality in 40–60 keV MEI (+) of visually hypoattenuating PDAC and in 40–50 keV MEI (+) of visually isoattenuating PDAC were significantly higher than those in PEI (*p* < 0.05). 40–50 keV MEI (+) provided significantly higher subjective scores for arteries and veins, compared to PEI (*p* < 0.05). There were no differences for all criteria between MEI (+) at 40 keV and 50 keV (*p* > 0.05). There was moderate-to-good interobserver agreement for all images at MEI (+) and PEI (kappa = 0.60–0.80).

**Table 3:** Qualitative analysis

	Hypoattenuating tumor							Isoattenuating tumor						
	40 keV	50 keV	60 keV	70 keV	80 keV	PEI	Kappa	40 keV	50 keV	60 keV	70 keV	80 keV	PEI	Kappa
Tumor conspicuity	*4.8 ± 0.4	*4.6 ± 0.5	*3.9 ± 0.4	3.3 ± 0.5	2.7 ± 0.5	3.4 ± 0.6	0.71	*3.8 ± 0.4	*3.6 ± 0.4	3.2 ± 0.3	2.8 ± 0.4	2.6 ± 0.4	2.9 ± 0.3	0.60
Tumor margin sharpness	*4.7 ± 0.4	*4.3 ± 0.7	*3.6 ± 0.5	2.9 ± 0.6	2.5 ± 0.4	3.1 ± 0.5	0.68	*3.7 ± 0.4	*3.5 ± 0.4	3.2 ± 0.4	2.7 ± 0.4	2.4 ± 0.4	2.7 ± 0.4	0.66
Image quality	*4.0 ± 0.3	*4.6 ± 0.5	*3.4 ± 0.5	3.2 ± 0.4	2.8 ± 0.4	3.2 ± 0.3	0.67	*3.8 ± 0.3	*4.4 ± 0.6	3.5 ± 0.5	3.2 ± 0.3	2.9 ± 0.3	3.2 ± 0.4	0.69
Depiction of arteries	*5.0 ± 0	*4.8 ± 0.4	4.3 ± 0.5	3.6 ± 0.5	3.2 ± 0.4	4.3 ± 0.6	0.80	*5.0 ± 0	*4.8 ± 0.4	4.2 ± 0.3	3.6 ± 0.5	3.2 ± 0.4	4.2 ± 0.4	0.71
Depiction of veins	*4.5 ± 0.5	*4.2 ± 0.6	3.5 ± 0.5	3.2 ± 0.3	2.5 ± 0.5	3.4 ± 0.4	0.70	*4.6 ± 0.5	*4.3 ± 0.4	3.6 ± 0.4	3.2 ± 0.4	2.7 ± 0.4	3.6 ± 0.5	0.70

Data are expressed as mean ± SD

The arteries include celiac trunk, common hepatic artery, and superior mesenteric artery. The veins include portal vein and superior mesenteric vein

*MEI ( +) > PEI in the qualitative image quality (*p* < 0.05)

## Discussion

Our study demonstrated that low keV MEI (+) could provide significantly better subjective and objective image quality, consistency of tumor measurements and tumor conspicuity compared to PEI, in patients with PDAC. The best tumor contrast and CNR values were obtained at 40-keV MEI (+) and image quality at 50-keV MEI (+). Notably, low-keV MEI (+) and iodine maps could also improve the diagnostic usability of visually isoattenuating PDAC, possibly enabling the early diagnosis.

In some prior investigations, researchers have suggested that low-keV MEI (+) can increase image quality and tissue contrast in pancreatic lesions [10, 12, 16], to improve tumor contrast. Beer et al. reported that in the dual-source DECT studies of 45 patients with PDAC, subjective and objective image quality at 40–50 keV MEI (+) were better than the PEI, and Nagayama et al. showed that tumor contrast and CNR of tumor were peaked at 40 keV without a relevant increase in image noise [10, 12]. Our study further confirmed their observations using a much larger patient cohort. In our study, the tumor contrast and CNR of tumor at 40-keV MEI (+) were about threefold and twofold higher than those of PEI. Similarly, the CNR of peripancreatic blood vessels at 40-keV MEI (+) increased by 91.98–102.31% compared with PEI. This further suggests that low-keV MEI (+) may help not only identify and diagnose tumor but also facilitate accurate local staging and determination of surgical resectability [19].

Image noise was an important measure to affect the image quality and tumor conspicuity. In our study, 40–60 keV MEI (+) had significantly higher but qualitatively acceptable image noise and 40–50 keV MEI (+) provided best subjective image quality. In addition, compared with standard monoenergetic imaging technique, a new image-based monoenergetic imaging technique for third-generation DECT has been investigated to acquire superior noise properties [20]. Moreover, an advanced modeled iterative reconstruction and tin pre-filtration for the high-kV tube were also employed to decrease the image noise. In addition, taking the SNR level into consideration is also necessary, although image noise is constantly implemented as an image quality indicator [13]. If the absolute noise level is clinically acceptable, increasing the SNR level could ensure the improvement of image quality. In our study, the SNR of pancreas at 40–60 keV MEI (+) increased by 13.79–48.28% compared with PEI. This suggested that although noise level increased in lower energies, the net benefits of improved tissue contrast seemed to exceed this deficiency [10].

Accurate measurement of tumor diameters was crucially important for tumor staging and evaluating treatment response such as radiotherapy [7, 21]. A previous study reported that reproducibility in tumor measurements at DECT image series (50 and 70 keV) was higher than the conventional 140 kVp images [6]. In our study, 40–50 keV MEI (+) demonstrated higher reproducibility of measurements than the higher energy images and PEI. This could be attributed to the improvement in tumor contrast and margin sharpness at low-keV MEI (+); therefore, the observers could distinguish the border between tumor and adjacent parenchyma and measure the tumor size more consistently [5].

Pathologically, visually isoattenuating PDAC had different characteristics, which composed of fewer cancer cellularity, more acinar tissue and loose fibrous stroma, and less amounts of mucin and necrosis, compared with usual PDAC [2]. The histopathological finding of visually isoattenuating PDAC may indicate the early change in the pathological progression of pancreatic cancers. Therefore, it may be one of the reasons that patients with visually isoattenuating PDAC have a better prognosis than those of usual PDAC. The frequency of visually isoattenuating PDAC in our study was consistent with previous investigations (5–17.5%) [2, 4, 22, 23]. Moreover, the quantitative analysis that the attenuation difference between tumor and the adjacent parenchyma was within 15 HU, further supported our visual assessment of isoattenuation PDAC. Previous studies suggested that an attenuation difference of less than 10–15 HU was generally difficult to be observed visually [2, 4]. In our study, we found that the highest tumor contrast and CNR of visually isoattenuating PDAC were obtained at 40-keV MEI (+), which were approximately fourfold and twofold higher than those of PEI. This suggested that low-keV MEI (+) could also improve the tumor conspicuity of visually isoattenuating PDAC and may allow the tumor to be identified earlier.

Besides the improvement of morphological details provided by low-keV MEI (+), DECT can assess iodine distribution in tissues. Quantifying iodine amount provided indirect indication of the underlying tissue microvascular environment, which corresponded to the degree of tissue perfusion at a specific point in time, and thus reflected the vascularity within the tumor [24]. Aslan et al. and McNamara et al. have shown that the NIC of tumor was lower than that of pancreatic parenchyma [3, 25]. However, the technique of this iodine quantification analysis was scantly applied in imaging of visually isoattenuating PDAC. In the current investigation, we observed that the NIC value of visually isoattenuating PDAC was also significantly lower than the parenchyma. Moreover, the NIC value of tumor in the visually isoattenuating PDAC was higher than that in the hypoattenuating PDAC. One possible explanation may be that visually isoattenuating PDAC was pathologically characterized by fewer cancer cellularity and looser fibrosis, compared with hypoattenuating PDAC [25, 26].

Our study had some potential limitations. First, this is a retrospective single-center study including a relatively small sample size, especially the patients with visually isoattenuating PDAC. Studies with large-scale populations are necessary to confirm the present result. Second, we focused on consistency of tumor measurements between observers in our study, but did not compare with measurements of the tumor specimens upon surgical resection. Surgical and pathology-correlated investigations may provide a clearer perspective on this study. Third, the mean diameter of tumors was larger than 2 cm, meaning that the results may not apply to smaller or early tumors. Finally, we did not compare the tumor detectability between MEI (+) images and PEI, rather focused on comparing the subjective and objective image parameters and tumor conspicuity. However, these works can provide a basis for further diagnostic study.

In conclusion, the use of low-keV MEI (+) on DECT substantially improved the subjective and objective image quality in patients with PDAC. 40-keV MEI (+) provided the greatest tumor conspicuity, blood vessel opacification, and reproducibility of tumor measurements for assessing PDAC. And iodine maps yield significant discriminatory information between tumor and normal pancreatic parenchyma. Combining the low-keV MEI (+) and iodine maps may yield diagnostically adequate tumor conspicuity in visually isoattenuating PDAC.

## Data Availability

The datasets used and/or analyzed during the current study are available from the corresponding author on reasonable request.

## Abbreviations

DECT: Dual-energy CT
PDAC: Pancreatic ductal adenocarcinoma
MEI: Monoenergetic image
PEI: Polyenergetic image
NIC: Normalized iodine concentration
CNR: Contrast-to-noise ratio
SNR: Signal-to-noise ratio
HU: Hounsfield units
ROI: Region of interest
